# The influence of regional deprivation index on personal happiness using multilevel analysis

**DOI:** 10.4178/epih/e2015019

**Published:** 2015-04-16

**Authors:** Kil Hun Kim, Jin-Ho Chun, Hae Sook Sohn

**Affiliations:** 1 Ulsan Emergency Medical Support Center, National Emergency Medical Center, Ulsan, Korea; 2 Department of Preventive Medicine, Inje University College of Medicine, Busan, Korea

**Keywords:** Happiness, Deprivation, Multilevel analysis

## Abstract

**OBJECTIVES:**

The objective of the present study was to identify the factors that influence the happiness index of community residents, by considering personal and regional aspects, and to use as evidence of efforts for improvement of the happiness index.

**METHODS:**

The study was conducted based on information from 16,270 participants who met the data requirement among those who participated in the 2011 South Gyeongsang Community Health Survey. Of the factors that can influence the happiness index, socioeconomic characteristics, health behavior, morbidity, and healthcare use, social contact, and participation in social activities were classified as personal factors; for regional factors, data from the 2010 census were used to extrapolate the regional deprivation indices at the submunicipal-level (eup, myeon, and dong) in South Gyeongsang Province. The happiness index for each characteristic was compared to that for others via t-test and ANOVA, and multilevel analysis was performed, using four models: a basic model for identification of only random effects, model 1 for identification of personal factors, model 2 for identification of regional factors, and model 3 for simultaneous consideration of both personal and regional factors.

**RESULTS:**

The mean happiness index was 63.2 points (64.6 points in males and 62.0 points in females), while the mean deprivation index was -1.58 points. In the multilevel analysis, the regional-level variance ratio of the basic model was 10.8%, confirming interregional differences. At the personal level, higher happiness indices were seen in groups consisting of males with high educational level, high income, high degree of physical activity, sufficient sleep, active social contact, and participation in social activities; whereas lower happiness indices were seen in people who frequently skipped breakfast, had unmet healthcare needs, and had accompanying diseases, as well as those with higher deprivation index.

**CONCLUSIONS:**

The study confirmed that the happiness index of community residents was influenced by not only personal aspects but also various regional characteristics. To increase the happiness index, interests at both personal and regional levels, as well as community emphasis on creating social rapport and engaging in selective efforts, are needed in vulnerable regions with relatively high deprivation index.

## INTRODUCTION

In modern society, life values are changing rapidly from preservation and extension of life to enhancement of well-being and happiness in life [1]. It has also been pointed out often in recent times that increases in income levels, until now typically measured by gross domestic product (GDP), do not always correspond with satisfaction and happiness in life [2], and that economic growth is an incomplete indicator of national welfare and well-being [3].

As such, with stronger recognition that economic prosperity is not directly related to quality of life, interest in not only materialistic, but also mental, quality of life and happiness are on the rise worldwide [4]. Accordingly, the importance of measuring subjective quality of life perceived by an individual has been highlighted, and the happiness index is receiving global attention as a new welfare index to replace economic growth [3]. Relating this to earlier events, in 1974 then King Jigme Singye Wangchuck of Bhutan announced that he would govern the country based on gross national happiness rather than GDP, and in 1981, the University of Michigan Institute for Social Research conducted the World Values Survey, which surveyed 1,000 to 2,000 citizens each from countries around the world with regard to happiness and life satisfaction and scored the results to compile subjective well-being rankings, which meant the happiness index was born [5]. Thereafter, in 2003, Rohwell, a British psychologist, and Cohen, a British life coach, announced their happiness formula, which reflected an individual’s personality traits, adaptability to cope with new situations, basic elements of survival, such as money, health, and sense of belonging, and high-level needs, such as personal pride and ambition. In 1999 in Canada, the Atkinson Charitable Foundation began a study on the Canadian Index of Wellbeing, eventually establishing 8 categories. In 2003, a research center at the Chinese University of Hong Kong (CUHK) announced the CUHK Hong Kong Quality of Life Index, which included 21 categories about society, economy, and environment.

The level of happiness is primarily influenced by personal characteristics, but it is also heavily influenced by the socio-physical environment and socioeconomic characteristics of residential areas. Within the municipal-level divisions (si, gun, and gu) of residential areas, there are administrative divisions with diverse socioeconomic backgrounds, and even for the same municipal- level division, there exist various submunicipal-level communities (eup, myeon, and dong) [6]. Because submunicipal-levels, as units of community, represent places where one’s own life directly influences or is directly influenced by the relationships among the community’s residents [7], the level of happiness should vary among submunicipal-levels. As such, since individuals form relationships and live with people within their area of residence, it is necessary to understand the influence of the residential area’s characteristics on individuals’ happiness [8].

Recently, there has been heightened interest in the influence of regional characteristics on individuals, and many studies have been conducted on the subject. Regional characteristics are being considered in various fields, and many studies have shown that regional characteristics affect the behavior or health conditions of individuals. Deprivation index, one of the representative variables that exhibit regional characteristics, is a numeric representation of quality of life based on housing environment, with a higher deprivation index signifying a poorer housing environment, and it is widely used as an index to reflect the socioeconomic level of a region. In each study of such a deprivation index, the sub-indices that comprise it vary slightly, and although many studies have used it as a regional characteristic, they have only considered its influence on health status, not satisfaction in life or happiness. We aimed to investigate the correlation between deprivation index and happiness index, which is closely correlated with health status.

Although there have been many studies on happiness index, few have considered regional factors and even those that have [9,10] were focused on city-level and county-level effects so rather than regional effects. Most studies on happiness index have examined specific groups or the outcomes of specific programs, based on one-dimensional analysis without distinguishing personal, group, or regional characteristics; no studies have considered region-specific variables for large groups. Moreover, when a one-dimensional analysis is performed on a data set that forms a hierarchical structure consisting of individuals and regions in which they live, the correlations among groups or individuals within a region are not been taken into account, which can yield inaccurate results. In addition, with a recent increase in the need to obtain individual and regional statistics, interest in small-area statistics format a submunicipal-level rather than municipal-level divisions is increasing day by day.

To address this need, the present study applied a modified deprivation index generated from various personal characteristics, obtained from the Korean Community Health Survey (CHS), and regional characteristics and personal characteristics at the submunicipal-level, obtained from the Population and Housing Census data from Statistics Korea, to decompose the happiness index for residents of South Gyeongsang Province into different levels by individual and region and performed a multilevel analysis to closely examine the factors that influence happiness and contribute to the enhancement of quality of life for the people of Korea.

## MATERIALS AND METHODS

### Participants and data

Based on 16,270 respondents from 20 cities and counties in South Gyeongsang Province who participated in the 2011 CHS, which was conducted for three months between August and November 2011, data were compiled for major observed variables and were used as a secondary source of data for the present study.

The measure of the dependent variable, happiness index, was one of the questions from the South Gyeongsang Province CHS conducted in 2008 by the Korea Institute for Health and Social Affairs. In the first stage, the institute reviewed precedent studies, domestic and foreign, on happiness index and happiness-related theories and worked with an expert advisory council to develop 10 categories regarding mental stability, family and marriage, personal relationships, community, daily life, demographic characteristics, economic stability, work, health, and residence. In the second stage, a total of 41 happiness indices, covering each of the 10 categories, were developed. Finally, in the third stage, a Delphi survey of the general population and the experts was conducted, to prioritize the 10 categories and the indices in each category. The result was a measurement tool that contained 9 categories and 21 total questions (Appendix 1) [10], with each question scored on a scale of 0 to 10 points. The sum of the scores for all questions in a category was converted to a category happiness index with a maximum of 10 points, and the sum of the 21 total questions’ scores was converted to a overall happiness index with a maximum of 100 points.

For personal characteristics, variables used were those predicted to be related to happiness index by the 2011 CHS and by existing studies related to happiness index [9-13]: participants’ degrees of physical activity, frequency of skipping breakfast, unmet healthcare needs, number of diseases, average sleep time, social network connections, and participation in social activities (Appendix 2).

For regional characteristics, raw data from Statistics Korea’s survey of the entire South Gyeongsang Province in the 2010 Population and Housing Census was used to calculate submunicipal-level regional deprivation indices. Submunicipal-level was the smallest unit for all items in the Population and Housing Census, which included information about population, households, and housing for a total of 3,160,154 people.

In order to calculate the deprivation index, the Carstairs index [14], Townsend index [15], and the index of multiple deprivation (all originally developed for the UK)—existing deprivation indices that reflect community characteristics—and component indices from existing studies [16-18] were referenced. From these, “less than high school graduation rate (25-64 years old),” “rate of population 65 years or older,” and “rate of widowed or divorced (15 years or older)” were selected to represent the population category and “rate of non-home-ownership,” “rate of lease or rent,” “rate of overcrowdedness,” “rate of female head of household,” “rate of poor housing environment,” and “rate of single person household” were selected for the household category (Appendix 2).

The method of calculating the deprivation index was the standard one of obtaining the value of each variable and, to compensate for influence, converting to Z-score before summation.

Moreover, in the process of matching the personal characteristic variables and the deprivation index, Jisu-myeon in the city of Jinju and Ssangchaek-myeon in Hapcheon county were excluded from the analysis, because their community health survey data were missing. In addition, towns with population less than 10 were combined with nearby communities that had similar geographical traits. As a result, 304 of the 323 towns in South Gyeongsang Province were included in the analysis (Appendix 3).

### Statistical analysis

SAS version 9.2 (SAS Institute Inc., Cary, NC, USA) was used for the statistical analysis. To gain an understanding of the data, means were calculated and frequency, mean comparison, and correlation analysis were performed. To identify the factors that influence the level of happiness index, a multilevel analysis that simultaneously considered both personal-level and regional-level characteristics was performed. Multilevel analysis is a method that can be used to analyze how personal-level dependent variables are influenced by personal-level and regional-level variables; such an analysis is appropriate when relying on data with hierarchical structure, such as data on individuals in submunicipal-levels (eup, myeon, and dong.) The basic model in multilevel analysis, designed to confirm the basic data, tests whether the differences among the variance values are significant without inputting the variables and can extrapolate the variance ratio based on regional characteristics among all variances [19]. This approach is called intraclass correlation, with the intraclass correlation coefficient (ICC) calculated as the ratio of the regional variance to the sum of the two levels of variances. The study models are those to which personal-level and regional-level variables can be inputted either separately or together. The explanatory power of each of the study models can be estimated as the ratio of the variances explained by inputting the variables from the study model into each level variance of the basic model. For the analyses, the Glimmix procedure in SAS was used.

In the present study, four models were established for the multilevel analysis.

Basic model: This model, which did not include any of the personal-level or regional-level variables, was constructed to estimate interregional variance.

Model 1: This model was constructed with personal-level variables, such as sex, age, education level, monthly household income, frequency of skipping breakfast, degree of physical activity, number of diseases, unmet healthcare needs, social network connections, and participation in social activities.

Model 2: This model was constructed with regional-level variables of deprivation indices for each town.

Model 3: This model included both personal-level and regional-level variables.

All statistical significance was determined based on a significance level of 0.05.

## RESULTS

### Participant characteristics and happiness index levels

#### Demographics, health status, and healthcare use

The participants included more females (55.8%) and the mean age was approximately 53 years. In terms of education level, the highest frequency was high school graduate (32.5%), while the average monthly household income was approximately 2.46 million Korean won (KRW). The rate of performing moderate or greater physical activity was 29.9%, the rate of skipping breakfast was 14.8%, and the average sleep time per day was 6.6 hours. Based on lifetime medical diagnoses, disease rates were 22.2% for hypertension, 7.9% for diabetes, and 16.9% for arthritis, with each participant having an average of 0.5 diseases. The rate of unmet healthcare needs was 16.8%.

The mean happiness index was significantly higher in males (64.6 points) than in females (62.0 points, p<0.001), and the overall happiness index was higher for individuals who had attained higher education levels (p<0.001).

Age and happiness index showed a significantly negative correlation (r=-0.201, p<0.001), whereas monthly household income and happiness index showed a significantly positive correlation (r=0.233, p<0.001).

The mean happiness index for the group engaging in moderate or greater physical activity (64.6 points) was significantly higher than that for the group that does not engage in such activity (62.6 points, p<0.001), while the mean happiness index was significantly lower for the group that skipped breakfast (62.4 points) than for the group that did not do so (63.3 points, p=0.002). The mean happiness index was significantly lower for the group with unmet healthcare needs (58.8 points) than for the treated group (64.0 points, p<0.001).

Sleep time and the happiness index showed a significantly positive correlation (r=0.102, p<0.001), whereas the number of diseases and the happiness index showed a significantly negative correlation (r=-0.164, p<0.001) (Table 1).

#### Socio-physical environment

With regard to social network contact of one or more times per week, surprisingly, contact with friends occurred with the smallest proportion of people (47.3%), while the proportions of people who had contact with relatives and neighbors were 49.6% and 69.0%, respectively. With regard to participation in social activities one or more times per month, 20.2% of people participated in religious activities, 59.0% in social activities, 23.3% in leisure activities, and 6.7% in community service activities.

The mean happiness index was significantly higher for the relatives contact group (64.5 points) than for the non-contact group (61.9 points, p<0.001), while the mean happiness index was significantly higher for the neighbor contact group (63.9 points) than for the non-contact group (62.8 points, p<0.001) and the mean happiness index was significantly higher for the friends contact group (65.7 points) than for the non-contact group (60.9 points, p<0.001).

The mean happiness index was significantly higher for the group participating in religious activities (65.2 points) than for the non-participation group (62.6 points, p<0.001), while the mean happiness index was significantly higher for the group participating in social activities (65.9 points) than for the non-participation group (59.2 points, p<0.001). The mean happiness index was significantly higher for the group participating in leisure activities (69.5 points) than for the non-participation group (61.3 points, p<0.001), while the mean happiness index was significantly higher for the group participating in community service activities (70.1 points) than for the non-participation group (62.7 points, p<0.001) (Table 2).

### Multilevel analysis results

In the basic model of the multilevel analysis for identification of factors that can influence the happiness index, the personal-level variance—the variance generated by personal differences among the participants—was 159.51, and the regional-level variance—the variance generated by differences among regions—was 19.351, showing a statistically significant difference. This result showed that interregional differences existed in the personal happiness index. In other words, a region can influence a person’s happiness index. Regional-level variance accounted for 10.8% of the total variance in happiness index, calculated by ICC=19.351/(19.351+159.51)=0.108.

In model 1, all variables except age had a significant influence on the happiness index, and their variances accounted for 18.7% of personal-level variance, (159.51-129.75)/159.51=0.187, and 24.8%, (19.351-14.546)/19.351=0.248, of regional-level variance. This result indicated that regional-level differences were explained by personal-level variables.

In model 2, a higher deprivation index was correlated with a significantly lower happiness index. Adding the regional-level variable, deprivation index, to model 1, explained 26.3%, (19.351-14.262)/19.351=0.263, of regional-level variance.

In model 3, the same influences of the independent variables from models 1 and 2 were seen. Higher happiness index was associated with being a male, being highly educated, having high income, engaging in moderate or high-intensity physical activity, getting sufficient sleep, having more social contact with relatives, neighbors, and friends, and actively participating in religious, social, leisure, and community service activities (p<0.001), whereas skipping breakfast, having unmet healthcare needs, and having more diseases meant a lower happiness index (p<0.001). Moreover, as the deprivation index increased, the happiness index decreased (p=0.025).

Through the processes above, the personal-level variance explained in the final model, model 3, was 18.7%, (159.51-129.75)/159.51=0.187, and the regional-level variance was 26.2%, (19.351-14.277)/19.351=0.262. In comparison to model 1, although 24.8% of regional-level variance was explained by personal-level variables, inclusion of the regional-level variables increased the explanatory power of the model by 1.4%. As such, interregional differences in happiness index were influenced by the deprivation index, a regional-level variable, but also by the majority of personal-level variables. Based on examination of χ^2^ values, each model was determined to fit (Table 3).

## DISCUSSION

There have been claims that more attention should be paid to assessment of welfare and sustainability rather than pure economic numbers as indices for measuring the level of welfare and benefits of citizens. There is an increased opinion, domestically and abroad, that standards that can measure not only economic growth but also social advancement and happiness of citizens are needed. Since the United Nations World Happiness Report of 2012, the importance of the happiness index has been emphasized.

As such, the present study used data from the CHS to perform a multilevel analysis, incorporating both personal and regional levels, to identify the factors that influence the happiness index for residents of South Gyeongsang Province. The intent was to present evidence for prioritizing improvement-related efforts and investments.

The multilevel analysis of the happiness index showed personal-level and regional-level variances in the basic model of 159.51 and 19.351, respectively, and because the ICC was 10.8%, interregional differences in happiness index appeared significant; hence, there was sufficient evidence to include regional-level factors in the model.

In looking at regional-level influences, a higher deprivation index meant a lower happiness index. In a sense, this is an obvious result: although no studies have considered the influence of deprivation index on happiness index, study has reported that good overall health status lead to a higher happiness index [12]. The findings of the present study are consistent with the results of Heo et al. [20] who used those aforementioned studies to examine the influence of deprivation index on health, which is highly correlated with happiness index, and reported that more experiences in socioeconomic deprivation lead to lower subjective health levels.

The present study confirmed that deprivation index, a regional-level characteristic, had an effect on the happiness index. This result showed the need to consider a region’s environmental factors when attempting to improve the happiness index of its residents.

With regard to personal-level influences, the happiness index was higher for males than for females. This result is consistent with studies by Kim & Han [9] and Abdel-Khalek [21] but is contrary to studies by Kim et al. [10] and Piqueras et al. [13], which showed higher happiness index for females. Moreover, studies by Vera-Villarroel et al. [22] and Choi & Moon [23] showed no gender-based differences in happiness index. However, as age increases, the happiness index generally decreases, so varying study results with regard to gender can be attributed to the age distribution of the study data. In other words, age is believed to affect the study results.

Although not statistically significant, an increase in age meant lower happiness index in the present study. This is consistent with studies by Vera-Villarroel et al. [22] and Choi & Moon [23], but slightly different from another study [24] that reported that recently in Europe and welfare states not in Europe happiness decreased with increasing age until the age of late 40s, after which it increased again, giving a U-shaped happiness profile over time. This U-shaped profile is believed to result from those countries having stable social security for the elderly, whereas in Korea, due to customary support for children, preparation for social security for the elderly and aging population is weak.

A higher education level meant higher happiness index. This result is consistent with studies by Kim et al. [10] and Choi & Moon [23]. The relationship is believed to be a result of education level’s effect on personal confidence, occupation, and income level, which can also influence family and social life. Similarly, higher monthly household income, which is closely related to education level, also meant higher happiness index. This is consistent with the study results by Campbell [25] and Diener et al. [26]. However, even with similar income, the relative values can vary, depending on individual circumstances, causing happiness index to vary [12]. In this way, Bhutan, with per capita income of only 1,200 US dollars, was ranked first in national happiness index as reported by the New Economics Foundation in 2010.

In terms of health behavior, greater engagement in moderate or high-intensity physical activity and longer average sleep time lead to higher happiness index, while more frequent breakfast skipping lead to lower happiness index. This is consistent with the study results of Chu [12] and Piqueras et al. [13]. Engaging in physical activity, which fundamentally requires good health, is believed to build strength and resistance to diseases, take one’s mind off tiring daily life, relieve stress, and provide a feeling of self-satisfaction. Eating breakfast and securing sufficient sleep contribute to physical and mental health by providing energy for daily activities, relieving fatigue from the previous day, and strengthening immune functions.

With regard to illnesses and healthcare use, having unmet healthcare needs and a greater number of accompanying diseases meant a lower happiness index. Such health-related problems have obvious results and are also related to economic problems.

As for socio-physical environmental characteristics, more contact with relatives, neighbors, and friends meant higher happiness index, as did more participation in religious, social, leisure, and community service activities. These findings are consistent with studies that reported participation in community service elevates subjective happiness [27,28] and religious and social activities [28] increase happiness index, as well as a study [29] that indicated leisure activities increase happiness index. In the culture of Korea group life is important: pride and satisfaction of participating in social activities is believed to lead to enhanced psychological stability and bonding, which increases subjective wellness and the happiness index. However, the present study’s univariate analysis showed that more frequent contact with neighbors resulted in lower happiness index. In contrast to contacts with relatives and friends, which are comfortable encounters based on blood ties, friendship, or familiarity, encounters with neighbors require a certain degree of etiquette, even with those known for a long time. These encounters, such as monthly neighborhood meetings, may not even be voluntary. Moreover, amount of contact with neighbors increased rapidly from age 50 years onward, which lead to the conclusion that there may be a related age-based effect on happiness index; the multilevel analysis compensated for such an effect.

With regard to the models’ explanatory power, model 1, which included only personal-level variables, explained 24.8% of the regional-level variance. In model 3, which included both personal- and regional–level variables, the explanatory power increased by 1.4% to 26.2%. This indicated that even though regional-level deprivation index influenced happiness index, the effects of personal-level variables were greater. In other words, interregional differences in happiness index were explained, almost entirely, by personal-level variables, meaning that most interregional differences resulted from personal-level characteristics.

In the process of calculating the deprivation index, which was the regional variable in the present study, one limitation was the inability to obtain male unemployment rate and rate of not owning a vehicle—they are both important variables that were not available at the submunicipal-level. As such, the raw data from the South Gyeongsang Province 10% sample survey (for which city and county were the smallest units) of the Population and Housing Census were used to perform a comparative analysis of the deprivation index from the study by Shin et al. [18] and the deprivation index from the present study; after checking the deprivation index’s reliability in this way, the present study was continued. Furthermore, although there was a question in the CHS about the length of residence in the corresponding area, differences based on regional characteristics can be seen within the same municipal-level divisions; hence, a more thorough assessment, with more detailed data, was not performed on the differential effects of regional characteristics based on length of residence in the community.

Despite these limitations, the present study was a systematic analysis to identify the population characteristics of South Gyeongsang Province based on sample data that well represented the region. As a study on happiness index, it is significant in that it is the first to include a multilevel analysis including both personal and regional characteristics. Although the effect of the regional variable, deprivation index, was relatively small compared to those of the personal characteristics, confirmation of the effect itself is significant. It is also meaningful that, to verify the influence of deprivation index more accurately, deprivation indices were derived at the submunicipal-level (eup, myeon, and dong).

It is hoped that, in the future, developing additional indices that can better reflect regional characteristics and incorporating the length of residence in the corresponding region can provide more accurate assessments.

Happiness index of community residents is influenced by not only personal characteristics but also regional characteristics. In a data analysis with such a hierarchical structure, multilevel analysis is required. Enhancement of happiness index and improved quality of life requires not only personal-level but also regional-level efforts, and for vulnerable areas—those with relatively high deprivation index—efforts should be made to create social rapport and engage in selective efforts.

## Figures and Tables

**Table 1.** Happiness index levels based on demographics, health status, and healthcare use

Characteristic	Classification	n (%)	Happiness index		
			Mean±SD	Correlation coefficient	p-value
Sex	Male	7,192 (44.2)	64.6±13.0		<0.001^1^
	Female	9,078 (55.8)	62.0±13.3		
Age (yr)	19-29	1,415 (8.7)			
	30-39	2,537 (15.6)			
	40-49	2,951 (18.1)			
	50-59	3,307 (20.3)			
	60-69	2,716 (16.7)			
	≥70	3,344 (20.6)			
	Mean±SD	53.1±16.8		-0.201	<0.001^2^
Education level	No education	2,596 (16.0)	55.9±13.4^3^		<0.001^4^
	Primary school	2,958 (18.2)	60.9±12.8^5^		
	Middle school	2,048 (12.6)	62.6±13.1^1^		
	High school	5,295 (32.5)	65.1±12.6^2^		
	College or higher	3,373 (20.7)	68.1±11.8^4^		
Monthly household income (×10^4^ KRW)	≤100	5,539 (34.0)			
	101-200	3,194 (19.6)			
	201-300	3,011 (18.5)			
	301-400	1,791 (11.0)			
	≥401	2,735 (16.8)			
	Mean±SD	245.7±235.8		0.233	<0.001^2^
Moderate or high-intensity physical activity^3^	No	11,405 (70.1)	62.6±13.5		<0.001^1^
	Yes	4,865 (29.9)	64.6±12.7		
Skipping breakfast^5^	No	13,868 (85.2)	63.3±13.3		<0.002^1^
	Yes	2,402 (14.8)	62.4±13.3		
Sleep time (hr)	Mean±SD	6.6±1.3		0.102	<0.001^2^
Disease diagnoses by doctor during lifetime	Hypertension	3,616 (22.2)			
	Diabetes	1,291 (7.9)			
	Arthritis	2,748 (16.9)			
No. of diseases	Mean±SD	0.5±0.7		-0.164	<0.001^1^
Unmet healthcare needs	No	13,533 (83.2)	64.0±13.0		<0.001^1^
	Yes	2,737 (16.8)	58.8±14.0		

SD, standard deviation; KRW, Korean won.

1 Statistical analysis conducted by t-test.

2 Pearson’s correlation coefficient.

3 In the past week, engaged in high-intensity physical activity for at least 20 minutes per day for 3 or more days or moderate physical activity for at least 30 minutes per day for 5 or more days.

4 Statistical analysis conducted by ANOVA.

5 In the past week, skipped breakfast 5 or more times.

**Table 2.** Happiness index levels based on socio-physical environment

Characteristic	Classification	n (%)	Happiness index	
			Mean±SD	p-value
Contact^1^ with relatives	No	8,207 (50.4)	61.9±13.5	<0.001^2^
	Yes	8,063 (49.6)	64.5±12.9	
Contact^1^ with neighbors	No	5,042 (31.0)	63.9±13.1	<0.001^2^
	Yes	11,228 (69.0)	62.8±13.3	
Contact^1^ with friends	No	8,574 (52.7)	60.9±13.4	<0.001^2^
	Yes	7,696 (47.3)	65.7±12.6	
Participation^3^ in religious activities	No	12,827 (78.8)	62.6±13.2	<0.001^2^
	Yes	3,443 (21.2)	65.2±13.5	
Participation^3^ in social activities	No	6,678 (41.0)	59.2±14.0	<0.001^2^
	Yes	9,592 (59.0)	65.9±12.0	
Participation^3^ in leisure activities	No	12,488 (76.8)	61.3±13.1	<0.001^2^
	Yes	3,782 (23.3)	69.5±11.8	
Participation^3^ in community service activities	No	15,177 (93.3)	62.7±13.3	<0.001^2^
	Yes	1,093 (6.7)	70.1±11.5	
Deprivation index	Mean±SD	-1.58±5.01	-0.164	<0.001^4^

SD, standard deviation.

1 Contact is defined as one or more times per week.

2 Statistical analysis conducted by t-test.

3 Participation is defined as one or more times per month.

4 Pearson’s correlation coefficient.

**Table 3.** Summary of factors affecting happiness index

Characteristic	Classification	Basic model	Model 1		Model 2		Model 3	
			Estimate	p-value	Estimate	p-value	Estimate	p-value
Sex	Male		0.565	0.004			0.580	0.003
Age			0.010	0.271			0.011	0.249
Education level			1.600	<0.001			1.582	<0.001
Monthly househdd income			0.006	<0.001			0.006	<0.001
Moderate or high-intensity physical activity			0.956	<0.001			0.962	<0.001
Skipping breakfast^1^			-2.486	<0.001			-2.488	<0.001
Mean sleep time			0.706	<0.001			0.710	<0.001
Unmet healthcare needs			-4.377	<0.001			-4.383	<0.001
No. of diseases			-0.620	<0.001			-0.622	<0.001
Social network	Relatives		2.354	<0.001			2.370	<0.001
	Neighbors		1.286	<0.001			1.345	<0.001
	Friends		1.680	<0.001			1.675	<0.001
Participation in social activities	Religious		1.585	<0.001			1.576	<0.001
	Social		3.489	<0.001			3.470	<0.001
	Leisure		3.676	<0.001			3.663	<0.001
	Community service		1.723	<0.001			1.740	<0.001
Deprivation index					-0.422	<0.001	-0.109	0.025
Random effect								
Personal-level variance		159.51^***^	129.75^***^		159.55^***^		129.75^***^	
Regional-level variance		19.35^***^	14.55^***^		14.26^***^		14.28^***^	
Intraclass correlation coefficient		0.108	0.101		0.082		0.099	
χ^2^		Reference	3,353^***^		65^***^		3,354^***^	
R^2^ (personal-level)		Reference	0.187		0.000		0.187	
R^2^ (regional-level)		Reference	0.248		0.263		0.262	

1 In the past week, skipped breakfast 5 or more times.

* p<0.05,

** p<0.01,

*** p<0.001.

## Appendix 1. Happiness index survey questions

**Table:**

Subcategory	Survey question
A. Psychological stability	1. How much self-esteem do you have?
	2. How would you rate your positive values and emotional state?
	3. How satisfied are you with your appearance?
B. Family/marriage	4. How satisfied are you with your family life or married life?
	5. How satisfied are you with your sex life?
	6. How satisfied are you with the birth and development of your children?
C. Personal relationships	7. How satisfied are you with your family members?
	8. How satisfied are you with your friends and colleagues?
	9. How satisfied are you with regard to receiving positive acknowledgement from other people?
D. Community	10. How satisfied are you with your community environment and infrastructure?
E. Daily life	11. How satisfied are you with leisure and rest?
	12. How satisfied are you with sleep (quantity and quality)?
F. Economic stability	13. How much of your financial (income) goals (gains and accumulation) do you feel you have achieved?
	14. How capable are you of being able to buy or have whatever you want, whenever you want?
G. Work/occupation	15. How fulfilling is your work?
	16. How closely does your current work match the work you wanted to do?
	17. How satisfied are you with your salary and work environment?
H. Health	18. How would you rate your health status?
	19. How would you rate your family members’ health status?
	20. To what degree do you engage in regular physical exercise?
I. Residence	21. How satisfied are you about ownership and quality of your residence?

Source from Kim et al. Study on determinants of happiness and happiness index in Koreans. Seoul: Korea Institute for Health and Social Affairs; 2008 [10].

## Appendix 2. Personal and regional characteristic variables

**Table:**

Category	Variable name	Definition
Personal characteristics		
Exercise and physical activity	Moderate or greater physical activity	Proportion of people who, for the past week, engaged in high-intensity physical activity^1^ for at least 20 minutes per day on 3 or more days per week or moderate physical activity^2^ for at least 30 minutes per day on 5 or more days per week
Nutrition	Skipping breakfast	Proportion of people who skipped breakfast 5 or more times in the past week
Mental health	Mean sleep time	Mean sleep time per day
Illness	Doctor diagnosis of hypertension, diabetes, or arthritis during lifetime	Percentage of people who have been diagnosed with hypertension, diabetes, or arthritis by a doctor
Healthcare use	Unmet healthcare needs	Percentage of people who, in the past year, did not receive the healthcare they needed
Socio-physical environment	Rate of social network contact (relatives, neighbors, or friends)	Percentage of people who engage in social network contact (relatives, neighbors, or friends) at least once per week
	Rate of participation in social activities (religious, social, leisure, or community service)	Percentage of people who regularly participate in social activities (religious, social, leisure, or community service) at least once per month
Regional characteristics		
Deprivation index	Rate of education level at or below high school graduation	Percentage of people aged 25-64 years who have education level at or below high school graduation
	Rate of non-home-ownership	Percentage of households that do not own a home
	Rate of lease or rent	Percentage of households that lease or rent their current residence
	Rate of overcrowdedness	Percentage of households with ≥1.5 persons living together in a room
	Rate of 65 years and older	Percentage of the population who are aged 65 years or older
	Rate of female head of household	Percentage of households with a female as the head
	Rate of widow and divorce	Percentage of those aged 15 or older who have been widowed or divorced
	Rate of poor living conditions	Percentage of households whose residence does not have its own kitchen, running water, hot water bath, or flush toilet
	Rate of one-person households	Percentage of households that consist of only one person

1 High-intensity physical activity severely fatigues the body or makes the body very short-winded.

2 Moderate physical activity slightly fatigues the body or makes the body slightly short-winded.

## Appendix 3. Towns combined or excluded from the study

**Table:**

Category	Municipal-level divisions	Towns
Combined	Gimhae-si	Saengnim-myeon and Sangdong-myeon; Hoehyeon-dong and Boowon-dong
	Changwon-si and Masan-gu	Gapo-dong, Gusan-myeon, and Hyun-dong; Odong-dong and Happo-dong
	Uiryeong-gun	Bongsu-myeon and Burim-myeon
	Jinju-si	Geumgok-myeon, Moonsan-eup, and Jinsung-myeon; Naedong-myeon, Jeongchon-myeon, Sabong-myeon, Ilbanseong-myeon, and Ibanseong-myeon; Daegok-myeon and Micheon-myeon; Kangnam-dong and Chilahm-dong; Joongang-dong and Bongahn-dong
	Changwon-si and Jinhae-gu	Joongang-dong and Chungmu-dong
	Hapcheon-gun	Chogye-myeon and Jeokjung-myeon
Excluded	Jinju-si	Jisu-myeon
	Hapcheon-gun	Ssangchaek-myeon
