# Supplementary material for: The influence of regional deprivation index on personal happiness using multilevel analysis
Source: Epidemiol Health. 2015 Apr 16;37:e2015019. doi: 10.4178/epih/e2015019 (PMC4835710; doi:10.4178/epih/e2015019)
Supplement: Supplementary file 1 [file epih-37-e2015019-supplementary.pdf]

3. 초록

목적: 지역사회 주민의 행복지수에 영향을 미치는 요인을 개인과 지역 측면까지 고려하여 파악하고 개선을 위한 노력의 근거 자료로 활용하고자 하였다.

방법: 연구대상은 2011년 경상남도 지역사회건강조사 참여자 중 자료가 충실한 총 16,270명 이었다. 행복지수에 영향을 미칠만한 요인으로 개인 측면은 사회경제적 특성, 건강행태, 이환 및 의료이용, 사회적접촉, 사회활동참여를 발췌하였고, 지역 측면은 2010년 인구주택총조사 자료를 이용하여 경상남도 읍면동별 지역 박탈지수를 산출하였다. 특성별 행복지수를 t-test와 ANOVA로 비교하였고, 임의효과만을 파악하기 위한 기초모델, 개인수준의 영향을 파악하기 위한 모델1, 지역수준의 영향을 파악하기 위한 모델2, 개인수준과 지역수준을 동시에 고려한 모델3의 총 4가지 모델로 다수준분석을 수행하였다.

결과: 대상은 남자 7,192명, 여자 9,078명으로 평균 연령은 53.1세였다. 행복지수 평균은 63.2점(남자 64.6점, 여자 62.0점), 박탈지수 평균은 -1.58점 이었다. 다수준분석 결과 기초모델의 지역수준 분산 비율이 10.8%로 나타나 지역 간에 차이가 있음을 확인하였다. 개인 측면에서는 남자, 고학력, 고소득, 더 많은 신체활동, 충분한 수면, 활발한 사회적접촉, 사회활동 참여 군에서 행복지수가 높았으며, 반면 아침 결식, 미충족의료, 동반질환수가 많은 경우와, 박탈지수가 높을수록 행복지수가 낮았다. 분산의 설명력은 개인수준 18.7%, 지역수준 26.2%였다.

결론: 지역 주민의 행복지수는 개인 측면뿐만 아니라 다양한 지역 특성에도 영향을 받음을 확인하였다. 행복지수 증대를 위해서는 개인 차원뿐만 아니라 지역 차원의 관심이 필요하겠으며, 상대적으로 박탈지수가 높은 지역, 즉 취약지역에 대한 지역사회의 공감대 형성과 선택적 노력이 강조되어야 할 것이다.

4. 중심단어

행복지수, 박탈지수, 다수준분석, 지역사회건강조사
Happiness index, Deprivation index, Multilevel analysis, Community health survey

5. 본문

서론

현대사회에서 삶에 대한 가치관은 기존의 생명보존과 수명연장 중심에서 웰빙 향상, 행복한 삶으로 급속히 변화하고 있다[1]. 그간 대표적으로 GDP 등으로 평가되어 온 소득 수준 증가는 삶의 만족감 및 행복감과 항상 일치하지는 않는다는 보고[2]와, 경제 성장은 국민의 후생과 복지 수준을 나타내는 지표로는 완전하지 못하다[3]는 지적이 최근 다수 제기되고 있다.

이렇듯 경제적 풍요가 삶의 질과 직결되는 것은 아니란 인식이 강해지면서, 전 세계적으로 물질적뿐만 아니라 정신적인 삶의 질이나 행복에 관심이 높아지고 있다[4]. 이에 따라 개인이 인지하는 주관적 삶의 질 측정의 중요성이 크게 부각되었으며, 경제 성장을 대신하는 새로운 후생지표로 행복지수가 세계의 관심을 끌고 있다[3]. 그로 일찍이 부탄에서는 1974년 Jigme Singye Wangchuck 전 국왕이 GDP가 아닌 국민들의 행복지수(Gross National Happiness)를 기준으로 나라를 통치하겠다고 발표했으며, 1981년 미국 미시간대 사회연구소가 주관하여 세계 각국 국민 1,000~2,000명에게 각자가 느끼는 행복도와 생활만족도 점수를 매기는 세계가치조사(World values survey)의 결과로 주관적 웰빙 지수(subjective well-being rankings), 즉 행복지수가 탄생되었다[5]. 이후 영국의 심리학자 Rohwell과 인생상담사 Cohen이 2003년에 개인의 성격특성 및 새로운 상황에 대처할 수 있는 적응력과 생존의 기본적 요소인 돈, 건강, 소속감, 그리고 개인의 자존심이나 야망과 같은 고차원적 욕구 등을 반영한 행복공식을 발표하였고, 캐나다에서는 1999년부터 왓킨슨 자선 재단(Atkinson Charitable Foundation)에서 국민의 삶의 질 지수(Canadian Index of Wellbeing)에 대해 연구를 시작, 8가지 영역을 확정 완성하였고, 홍콩에서는 2003년 중문대 연구센터에서 사회, 경제, 환경 등 21개 항목으로 홍콩 삶의 질 지수(CHUK Hong Kong Quality of Life Index)를 발표하였다.

한편, 행복수준은 일차적으로는 개인 특성에 영향을 받지만 이와 더불어 거주 지역의 사회물리적 환경, 사회경제적 특성 등에도 많은 영향을 받을 것이다. 그리고 거주지역의 시군구 내에는 다양한 사회경제적 배경을 가지는 행정구역이 있고, 같은 시군구라 하더라도 그 내부에는 다양한 읍면동의 지역사회가 존재하며[6], 지역사회 단위로서의 읍면동은 자신의 삶이 지역 사람들 사이의 관계에 직접적인 영향을 미치기도, 받기도 하는 장소이기 때문에[7], 동일 시군구 내에서도 하

위 단계인 읍면동별로 행복수준은 차이가 날 것이다. 이렇듯 개인은 거주지역의 사람들과 관계를 맺고 함께 살아가고 있으므로 거주지역의 특성이 미치는 영향을 이해하는 것이 필요하다[8].

최근 지역의 특성이 개인에게 미치는 영향에 대한 관심이 높아지고, 이에 대한 연구도 다수 이루어졌다. 다양한 분야에서 지역특성이 고려되고 있으며, 많은 연구들에서 지역특성이 개인의 행동이나 건강상태 등에 영향을 미치는 것으로 나타났다. 지역특성을 나타내는 많은 변수들 중 대표적인 박탈지수(Deprivation index)는 주거환경을 중심으로 삶의 질을 수치화 한 것으로, 박탈지수가 높을수록 주거환경이 좋지 않은 것이라 할 수 있고, 지역의 사회경제적인 수준을 반영하는 지표로 많이 활용되고 있다. 박탈지수는 연구마다 구성하는 하위지표들이 조금씩 다르고, 이를 지역특성으로 활용한 다수의 연구들이 있었으나, 삶의 만족감 또는 행복지수가 아닌 건강수준에 박탈지수가 미치는 영향을 고려한 연구만 이루어졌었다. 이에 건강수준과 밀접한 상관성이 있는 행복지수와 박탈지수간의 상관성이 있는지 알아보고자 하였다.

그 동안 행복지수에 대한 많은 연구들이 이루어져 왔지만 지역을 고려한 연구는 많지 않았으며 지역을 고려한 연구들[9,10] 등에서도 시군 단위까지의 연구였으며, 지역효과를 고려하지 않는 연구들이었다. 행복지수에 대한 기존의 연구들은 대부분 특정 집단이나 특정 프로그램의 결과를 대상으로 개인 특성과 집단이나 지역 특성의 구분 없이 일차원적인 분석들로만 연구되어 왔으며, 대규모 집단에 대한 지역 특성 변수를 고려한 연구는 없었다. 그리고 자료의 구조가 개인과 개인들을 포함하는 지역으로 이루어진 위계적인 구조를 가질 때 일차원적인 분석을 실시하게 되면, 집단이나 지역 내 개인들 사이의 상관성이 고려되지 않아 부정확한 결과가 도출될 수 있다. 또한 근래 개인과 지역근래 지역별통계 산출 필요성이 증대됨에 따라 시군구를 넘어 읍면동별(town-level) 단위의 소지역 통계에 대한 관심도 날로 높아지고 있다.

이에 본 연구는 지역사회건강조사로부터 확보 가능한 다양한 개인 특성 변수와, 통계청의 인구주택총조사로부터 확보 가능한 읍면동별 개인 및 지역 특성 변수로부터 생성한 보정 박탈지수(Modified Deprivation index)를 적용하여 경상남도 주민의 행복지수를 개인과 지역을 다른 수준으로 분리하여 다수준분석을 실시하고, 행복에 미치는 영향 요인을 구명하며 나아가 한국인의 삶의 질 향상에 일조하고자 하였다.

대상 및 방법

1. 연구대상 및 자료

2011년 8월부터 11월까지 3개월간 시행된 2011년도 지역사회건강조사 경상남도 20개 시군의 응답자 중 주요관측변수가 충실한 자료를 2차 자료원으로 이용하였으며, 총 대상자 수는 16,270명이었다.

종속변수인 행복지수 측정도구는 지역사회건강조사 경상남도의 선택 문항 중 하나로 2008년도 한국보건사회연구원에서 1단계로 행복지수에 관한 국내외 선행연구들과 행복관련 이론, 전문가 자문회의를 거쳐서 심리적 안정, 가족.결혼, 개인적 관계, 지역사회, 일상생활, 인구사회학적 특성, 경제적 안정, 일, 건강, 주거 10개의 영역을 개발하였다. 2단계로 각 영역별 행복지표 총 41개를 개발하였고, 마지막 3단계로 일반인 및 전문가를 대상의 델파이 조사를 통하여 10개 영역에 대한 우선순위와 각 영역내 지표들에 대한 우선순위와 영향정도를 분석하여 최종적으로 9개영역 총 21개 문항으로 개발된 측정도구이며**(부록 1**)[10], 각 문항에 대해 0~10점으로 응답을 하였다. 각 세부영역별 행복지수는 영역내 문항들의 합을 10점 만점으로 환산하였으며, 전체 행복지수는 총 21문항의 합을 100점 만점으로 환산하였다.

개인 특성 변수로는 2011년 지역사회건강조사에서 행복지수와 관련이 있을 것으로 예상되는 변수들로 기존의 행복지수에 관한 연구들[9-13]을 참고하여 중등도 이상 신체활동 실천율, 아침결식률, 필요의료서비스 미치료율, 질환개수, 평균수면시간, 사회적 연결망 접촉률, 사회활동 참여율 등을 활용하였다**(부록 2).**

지역 특성 변수로는 통계청에서 실시한 2010년 인구주택총조사의 경상남도 전수를 조사한 원자료를 이용하여 읍면동별 지역박탈지수를 산출하였다. 인구주택총조사의 전수항목은 읍면동이 최소단위로 제공되는 자료로 2010년에는 경상남도의 3,160,154명이 조사되었으며 자료제공범위는 크게 인구부분, 가구부분, 주택부분이다.

박탈지수를 산출하기 위해 지역사회 특성을 반영하는 기존의 지수인 카스테어즈지수[14], 타운젠트지수[15], IMD(Index of Multiple Deprivation(UK))와 기존의 연구들[16-18]의 구성지표를 참고하여 인구부분에서 ‘고졸미만 교육수준비율(25-64세)’, ‘65세 이상 인구율’, ‘사별 및 이혼율(15세 이상)’을 선정하였고, 가구부분에서 ‘주택미소유율’, ‘전월세율’, ‘과잉밀집률’, ‘여성 가구주율’, ‘열악

한 주거환경률', '1인 가구율'의 9개 변수를 최종적으로 선정하였다**(부록 2).**

박탈지수의 산출 방법으로는 가장 일반적으로 많이 사용되어진 방법으로 각 변수들의 값을 구하고, 영향력을 보정해 주기 위해 Z-score로 변환한 후 합산하는 방법을 사용하였다.

그리고 개인특성 변수들과 박탈지수를 매칭하는 과정에서 경상남도 323개의 읍면동 중에서 지역사회건강조사 자료에서 누락된 진주시 지수면, 합천군 쌍책면을 분석에서 제외하고, 조사대상자가 10명 미만인 읍면동은 지리적으로 성격이 비슷한 인접한 지역과 통합하여 최종적으로 304개의 읍면동을 대상으로 분석하였다**(부록 3).**

2. 통계분석

통계분석은 SAS (ver 9.2) 를 이용하여 분석하였으며, 자료의 개요를 파악하기 위해 빈도, 평균분석을 실시하였고, 특성에 따라 평균비교 및 상관분석을 실시하였다. 행복지수 수준에 영향을 미치는 요인을 파악하기 위해 개인수준특성과 지역수준특성을 동시에 고려한 다수준분석을 실시하였다. 다수준분석은 개인수준의 종속변수가 개인 및 지역수준의 변수들에 영향을 받는 것을 분석할 수 있는 방법으로, 본 연구자료와 같이 개인들이 읍면동이라는 지역수준에 포함되어 있는 위계적 구조의 자료를 분석하기에 적절한 방법이다. 다수준분석의 기초모델은 기초정보를 확인하는 과정으로 변수를 투입하지 않은 상태에서 개인수준과 지역수준의 분산 값의 차이가 유의한지를 검증하고, 전체 분산 중 지역특성에 의한 분산비율을 도출할 수 있다[19]. 이를 ICC (intra class correlation)라 하며, ICC는 두 수준의 분산을 합한 전체 분산으로 지역수준의 분산을 나눈 값으로 구할 수 있다. 연구모델은 개인수준 변수와 지역수준 변수를 각각 투입하는 모델과 모두 투입하는 모델이다. 연구모델에서의 설명력은 기초모델의 각 수준 분산에서 연구모델의 변수 투입으로 설명되는 각 수준 분산의 비율로 구할 수 있다. 분석에는 SAS의 Glimmix procedure를 이용하였다.

본 연구에서는 다수준분석을 위해 4가지 모델을 구축하였다.

기초모델 : 지역간의 변이를 추정하기 위해 개인수준과 지역수준 변수 모두를 포함하지 않은 모델을 구축하였다.

모델 1 : 개인수준 변수인 성별, 연령, 교육수준, 월가구소득, 아침결식, 중등도 이상 신체활동, 질환개수, 필요의료서비스 미치료, 사회적 연결망 접촉, 사회활동 참여로 모델을 구축하였다.

모델 2 : 지역수준 변수인 읍면동별 박탈지수로 모델을 구축하였다.

모델 3 : 개인수준 변수와 지역수준 변수를 모두 포함하여 모델을 구축하였다.

모든 통계적 유의성의 판단은 유의수준 0.05를 기준으로 하였다.

결과

1. 대상자 특성 및 특성별 행복지수 수준

(1) 인구사회학적, 건강행태, 이환 및 의료이용

성별 분포는 55.8%로 여자가 많았고, 평균 연령은 약 53세였다. 교육수준은 고졸 32.5%가 가장 많았으며, 월가구소득은 평균 약 246만원 이었다. 중등도 이상 신체활동 실천율은 29.9%, 아침결식률은 14.8%이었고, 하루 평균 수면시간은 6.6시간이었다. 평생 의사진단 경험률에서 고혈압은 22.2%, 당뇨병은 7.9%, 관절염은 16.9%이었으며, 평균 질환 개수는 0.5개, 필요의료서비스 미치료율은 16.8%이었다.

행복지수 평균은 남자(64.6점)가 여자(62.0점) 보다 유의하게 높았으며($p<0.001$), 교육수준이 높을수록 전체 행복지수가 높았다($p<0.001$).

연령과 행복지수의 상관관계는 유의한 음의 상관을 보였다($r=-0.201$, $p<0.001$). 월가구소득과 행복지수의 상관관계는 유의한 양의 상관을 보였다($r=0.233$, $p<0.001$) .

중등도 이상 신체활동 실천에 따른 전체 행복지수 평균은 실천군(64.6점)이 실천하지 않는 군(62.6점) 보다 유의하게 높았으며($p<0.001$), 아침결식에 따른 행복지수 평균은 결식군(62.4점)이 결식예방군(63.3점) 보다 유의하게 낮았으며($p=0.002$), 필요의료서비스 미치료에 따른 행복지수 평균은 미치료군(58.8점)이 치료군(64.0점) 보다 유의하게 낮았다($p<0.001$).

수면시간과 행복지수의 상관관계는 유의한 양의 상관을 보였으며($r=0.102$, $p<0.001$), 질환개수와 행복지수의 상관관계는 유의한 음의 상관을 보였다($r=-0.164$, $p<0.001$)**(Table 1).**

(Table 1)

(2) 사회물리적 환경

일주일에 한 번 이상 사회적 연결망 접촉률에서 친척은 49.6%, 이웃 69.0%, 친구 47.3%로 의외로 친구와의 접촉률이 가장 낮았다. 한 달에 한 번 이상 사회활동 참여율에서 종교활동은 21.2%,

친목활동 59.0%, 레저 활동 23.3%, 자선단체활동 6.7%이었다

친척과의 접촉에 따른 행복지수 평균은 접촉군(64.5점)이 미접촉군(61.9점) 보다 유의하게 높았으며 ($p<0.001$), 이웃과의 접촉에 따른 행복지수 평균은 접촉군(63.9점)이 미접촉군(62.8점) 보다 유의하게 높았고($p<0.001$), 친구와의 접촉에 따른 행복지수 평균은 접촉군(65.7점)이 미접촉군(60.9점) 보다 유의하게 높았다($p<0.001$).

종교활동 참여에 따른 행복지수 평균은 참여군(65.2점)이 미참여군(62.6점) 보다 유의하게 높았으며($p<0.001$), 친목도모활동 참여에 따른 행복지수 평균은 참여군(65.9점)이 미참여군(59.2점) 보다 유의하게 높았으며($p<0.001$), 레저활동 참여에 따른 전체 행복지수 평균은 참여군(69.5점)이 미참여군(61.3점) 보다 유의하게 높았고($p<0.001$), 자선단체활동 참여에 따른 행복지수 평균은 참여군(70.1점)이 미참여군(62.7점) 보다 유의하게 높았다($p<0.001$)**(Table 2).**

(Table 2)

2. 다수준분석결과

행복지수에 영향을 미치는 요인을 알아보기 위한 다수준분석결과로 기초모델에서 대상자 개인의 차이에 의해 발생하는 개인수준 분산은 159.51이고, 지역 간의 차이에 의해 발생하는 지역수준 분산은 19.351로 통계적으로 유의한 차이가 있었고, 이로 개인의 행복지수에 지역 간의 차이가 존재하며 지역이 개인의 행복지수에 영향을 미칠 수 있다는 것을 알 수 있었다. 행복지수의 전체 분산 중 지역수준의 분산이 차지하는 비율은 ICC=19.351/(19.351+159.51)=0.108로 10.8%였다.

모델 1에서는 연령을 제외한 모든 변수가 행복지수에 유의한 영향을 미치고 있었으며, 개인수준 변수들의 투입에 따른 분산 (159.51-129.75)/159.51=0.187, 18.7%가 추가로 설명되었고, 지역수준의 분산은 (19.351-14.546)/19.351=0.248, 24.8%가 설명되고 있어 지역수준의 차이가 개인수준 변수들에 의해 설명되고 있음을 알 수 있었다.

모델 2에서는 박탈지수가 높을수록 행복지수가 유의하게 낮았으며, 지역수준 변수인 박탈지수를 추가 했을 때 지역수준의 분산 (19.351-14.262)/19.351=0.263, 26.3%가 설명되었다.

모델 3에서는 모델 1, 2에서의 독립변수들의 영향과 같은 결과를 보였다. 행복지수는 남자, 고

학력, 고소득, 중등도 이상 신체활동, 충분한 수면을 취하는 군, 친척, 이웃, 친구의 사회적 연결망에 접촉할수록, 종교, 친목, 레저, 자선단체의 사회활동 참여가 활발할수록 높아졌으며($p<0.001$), 아침 결식, 미충족의료가 있을 때, 동반질환수가 많을수록 낮아졌고($p<0.001$), 박탈지수가 높을수록($p=0.025$) 낮아졌다.

이상의 과정을 거쳐 최종모델에서 설명된 개인수준분산은 (159.51-129.75)/159.51=0.187, 18.7%였고, 지역수준분산은 (19.351-14.277)/19.351=0.262, 26.2%였다. 모델 1과 비교 결과, 지역수준의 분산이 개인수준 변수들로 24.8%가 설명되었고 지역수준 변수를 추가함으로써 설명력이 1.4% 증가하였다. 이로써 행복지수의 지역 간의 차이는 지역수준 변수인 박탈지수에도 영향을 받지만 상당 부분 개인수준 변수들의 영향을 받고 있음을 관찰하였다. χ^2값으로 살펴본 결과 각 모델은 모두 적합하였다**(Table 3)**.

(Table 3)

고찰

국민의 후생과 복지 수준을 나타내는 지표로써 순수 경제적 수치보다 복지, 지속 가능성에 대한 평가에 더 주목해야 한다는 주장이 나오고 있다. 경제성장은 물론 사회 발전, 국민의 행복을 측정할 수 있는 기준이 필요하다는 여론이 국내외적으로 증대되고 있으며, 2012년 유엔 세계 행복 보고서 발표 이후 행복지수의 중요성이 강조되고 있다.

이에 본 연구는 지역사회건강조사 자료를 활용하여 경상남도 주민의 행복지수에 영향을 미치는 요인을 개인 차원 뿐만 아니라 지역차원까지 고려하여 다수준분석을 통해 파악하고 개선을 위한 노력과 투자의 우선순위 설정의 근거를 제시하고자 하였다.

전체 행복지수에 대한 다수준분석결과 기초모델의 개인수준 분산은 159.51, 지역수준 분산은 19.351이었으며 ICC값이 10.8%로 행복지수의 지역 간 차이가 유의하게 나타나 지역수준까지 모형에 포함할 근거가 충분하였다.

지역수준의 영향을 알아본 결과 박탈지수가 높을수록 행복지수는 낮았다. 이는 어떻게 보면 당연한 결과로 행복지수에 대한 박탈지수의 영향을 고려한 연구는 없었지만, 삶의 만족감과 건강수준은 밀접한 상관성이 있으며[20], 전반적 건강상태가 좋을수록 행복지수가 높다[12] 는 연구들을

바탕으로 행복지수와 상관성이 높은 건강에 대한 박탈지수의 영향을 살펴본 연구에서 사회경제적 박탈을 많이 경험할수록 주관적 건강수준은 낮아진다는 허종호[21]의 연구와 일치한다.

이렇듯 행복지수 수준에 지역수준 특성인 박탈지수가 영향을 미치는 것을 확인할 수 있었다. 행복지수 향상에 있어 지역의 환경적 요인들도 충분히 고려해야 할 필요성을 보였다.

개인수준의 영향은 성별에 따른 행복지수 수준은 남자가 여자보다 높았다. 이는 김명소[9], Abdel-khal다[22] 등의 연구와 일치하였고 김승권[10], Piqueras[13], 등의 연구는 여자가 더 높아 상이한 결과였으며 Vera-Villarroel[23], 최말옥[24] 등의 연구에서는 성별의 차이는 없었다. 일반적으로 연령이 높을수록 행복지수가 낮아지는데 성별에 따른 연구결과들이 다양한 것은 각 연구 자료의 연령분포에 기인하는 것으로 판단된다. 즉 연령에 따른 성별분포가 연구결과에 영향을 미치는 것으로 판단된다.

통계적으로 유의하진 않았지만 연령이 증가할수록 행복지수가 낮아졌다. 이는 Vera-Villarroel[23], 최말옥[24] 등의 연구와 일치하는 결과로 최근 유럽이나 복지국가에서 연령이 증가할수록 감소하다가 40대 이후에 다시 증가하는 U자형의 행복수준을 가지는 것[25] 과는 약간 다른 결과이다. 이런 결과는 유럽이나 복지국가에서는 안정된 노후보장이 이루어지지만 우리나라는 일반적 풍토인 자녀들을 위한 지원으로 인해 노후보장 준비와 인구고령화에 대한 대책이 미흡한데 의한 것으로 생각할 수 있다.

교육수준은 높을수록 행복지수가 높았다. 이는 김승권[10], 최말옥[24] 등의 연구결과와 일치하는 것으로 교육수준에 따라 개인의 자신감과 직업 및 소득수준의 차이가 나타나고, 그에 따른 가족 및 사회생활에도 영향을 주기 때문일 것이라 판단된다. 이와 같은 맥락으로 교육수준과 밀접한 관련이 있는 월가구소득은 많을수록 행복지수가 높았다. 이는 Campbell[26], Diener[27] 등의 연구결과와 일치한다. 하지만 비슷한 수입이라 하더라도 각자의 상황이나 여건에 따라 절대적 가치가 다를 수 있고 이에 따라 행복지수 또한 달라질 수 있다[12]. 이는 2010년 유럽신경제재단(NEF)에서 발표한 국가별 행복지수 1위가 1인당 국민소득 1200달러에 불과한 부탄이 차지한 맥락과 같다고 볼 수 있다.

건강행태에 따라서는 중등도 이상 신체활동을 할수록, 평균수면시간이 많을수록 행복지수가 높았고 아침을 결식할수록 행복지수가 낮았다. 이는 추홍규[12], Piqueras[13] 등의 연구결과와 일치

한다. 신체활동으로 강인한 체력을 기름으로써 질병에 대한 저항력을 키워 건강한 삶을 영위할 수 있고, 신체활동을 함으로써 지친 일상을 잊고 스트레스를 해소하며 자기만족감도 느끼기 때문이라 판단되며 근본적으로 중등도 이상의 신체활동을 하기에는 건강이 뒷받침 되어야 하기 때문이라 할 수 있겠다. 아침 결식예방과 충분한 수면시간 확보는 일상을 보낼 수 있는 에너지 공급과 전날의 피로를 풀어주고 면역기능을 강화하는 등 신체적, 정신적 건강에 기여하는 것으로 판단된다.

이환 및 의료이용에서는 필요의료서비스를 못 받으면, 동반질환개수가 많을수록 행복지수가 낮았다. 이는 건강상의 문제가 있는 것을 의미하므로 당연한 결과이며 필요의료서비스 미충족은 경제적인 측면과 관련이 있기 때문이다.

사회물리적 환경 특성별로는 친척, 이웃, 친구와 접촉할수록 행복지수가 높았고, 종교, 친목, 레저, 자선활동의 사회활동에 참여할수록 행복지수가 높았다. 이는 자선활동에 참여하면 주관적 행복감이 높다는 연구[28,29]와 종교활동, 친목활동을 할수록[29], 레저활동을 할수록 행복지수가 높다는 Mancini[30] 의 연구와 일치한다. 집단생활을 중요시 하는 우리나라의 의식구조상 사회활동의 참여로 그 활동에 대한 자부심과 만족감이 심리적 안정과 유대감의 증진으로 이어지고 주관적 안녕감, 행복지수를 높인 것으로 판단된다. 단변량 분석결과에서 이웃과는 오히려 접촉할수록 행복지수가 낮았는데, 친척, 친구와의 접촉은 혈육, 우정과 친분으로 이어진 편안한 만남인데 반해 이웃과는 오래 알고 지낸 사이여도 어느 정도 예의를 갖춰야 하기 때문인 것과 조사되어진 접촉이 반상회 같은 자의적인 만남이 아닐 수도 있는 것으로 판단된다. 또한 이웃과의 접촉하는 군이 50대부터 급격하게 증가되었는데 연령에 의한 영향도 있었을 거라 판단되며, 다수준분석에서는 연령이 보정된 결과가 나타난 것으로 볼 수 있다.

기초모델을 기준으로 변수들을 포함했을 때의 지역수준분산의 설명 정도에서 개인수준만을 포함한 모델1은 설명력이 24.8%였고, 개인수준과 지역수준까지 포함한 모델3은 설명력이 1.4% 증가한 26.2%였다. 이는 행복지수 수준에 있어서 지역수준의 박탈지수도 영향이 있었지만 개인수준 변수들의 영향이 더 컸다고 할 수 있다. 즉 행복지수의 지역 간의 차이가 개인수준 변수들에 의해 거의 설명되고 있는, 다시 말하면 지역 간의 차이가 상당부분 개인수준의 특성에 기인하여 발생한다고 볼 수 있다.

본 연구의 지역변수인 박탈지수를 산출하는 과정에서 읍면동별로 추출 가능한 변수들 중 중요할 것으로 여겨지는 남성 실업률과 자동차 미소유율을 구할 수 없음이 제한점으로 여겨졌다. 이에 남성 실업률과 자동차 미소유율을 구할 수 있는 인구주택총조사 경상남도의 10% 표본조사 원자료(최소단위 시군구)를 이용하여 기존의 신영전, 윤태호 연구[18]의 박탈지수와 본 연구의 박탈지수를 산출하여 비교 분석하여 그 신뢰도를 확인한 후 연구를 진행하였다. 더불어 지역사회건강조사에 해당지역에서의 거주기간을 묻는 문항은 있었지만, 같은 시군구 내에서도 지역 특성의 차이가 있을 수 있으므로 더 세분화된 자료를 근거로 주거지역에서 거주한 기간에 따른 지역 특성의 영향 차이를 충실히 평가하지 못한 점이 아쉽다.

이러한 제한점에도 불구하고 본 연구는 체계적인 분석을 통해서 경상남도의 모집단의 특성을 파악하고 이를 잘 반영할 수 있는 추출법을 적용한 표본자료를 사용하여 지역의 대표성을 최대한 반영하고, 행복지수의 연구에 있어서 개인특성을 포함하여 지역특성까지 고려하여 다수준분석을 실시한 처음 연구라는 적지 않은 의미를 지닌다. 비록 지역특성인 박탈지수의 영향은 개인특성들에 비해 상대적으로 크진 않았지만 영향이 있음을 확인한 것에 큰 의의를 부여하며, 행복지수에 지역특성인 박탈지수의 영향력을 보다 정확하게 확인하기 위해 지역구분을 읍면동 단위까지 분류하고 읍면동 박탈지수를 산출하여 사용한 점에서 의미가 있다.

향후 지역특성을 보다 잘 반영하는 추가 지표들의 개발과, 해당 지역의 거주기간을 보다 정확히 반영하는 노력을 통하여 이와 관련한 보다 정확한 평가를 내릴 수 있기를 희망한다.

결론

지역 주민의 행복지수는 개인 특성뿐만 아니라 지역 특성에도 영향을 받음을 확인하였다. 이처럼 위계적 구조의 자료 분석에서는 지역 특성까지 고려한 다수준분석이 필요할 것이다. 행복지수 향상과 삶의 질 개선을 위해서는 개인 차원뿐만 아니라 지역 차원의 개선 노력이 필요하겠으며, 상대적으로 박탈지수가 높은 지역, 즉 취약지역에 대한 지역 사회의 공감대 형성과 선택적 노력이 강조되어야 할 것 이다.

6. 참고문헌

1. Freed MM. Quality of life: The Physician's dilemma. Arch Phys Med Rehabil 1984;65(3):109-111.

2. 3RD OECD World Forum, Busan Korea. 2009.10.

3. Andrew Leigh. Growth Matters. Aurora Magazine, Issue 3. 2006.

4. Hwang MJ, Shim sj. Development of Happiness Index of Korea. The Korean Association for Survey Research 2008;9(3):93-117.

5. Hays RD, Morales LS. The RAND-36 measure of health-related quality of life. Ann Med 2001;33(5):350-357.

6. Kim CS, Yun SC, Kim HR, Khang YH. A Multilevel Study on The Relationship between the Residential Distribution of High Class (Power Elites) and Smoking in Seoul. J Prev Med Public Health 2006;39(1):30-38.

7. Kirst-Ashman, Karen K, Grafton H, Hull Jr. Generalist Practice with Organizations and Communities. Brooks/Cole. Belmont, CA. 2001

8. Robert SA. Community-Level Socioeconomic Status Effects on Adult Health. Journal of Health and Social Behavior 1998;39(1):18-37.

9. Kim MS, Han YS. The Development of Happiness Index for Korean. The Korean Association for Survey Research 2006;7(2):1-38.

10. Kim SK, Jang YS, Cho HS, Cha MS. Study of Determinants and Indicators of Happiness among Koreans. Korea Institute for Health and Social Affairs . 2008.12.

11. Ballas D, Dorling D. Measuring the impact of major life events upon happiness. Int Jr Epidemiol. 2007;36(6):1244-1252.

12. Chu HK. The Level of Happiness Index and Determining Factors [dissertation]. Gimhae: Inje University; 2005 (Korean).

13. Piqueras JA, Kuhne W, Vera-Villarroel P, Straten AV, Cuijpers P. Happiness and health behaviours in Chilean college students: A cross-sectional survey. BMC Public Health 2011;11:Article number 443.

14. Carstairs V, Morris R. Deprivation and health in Scotland. Health Bull (Edinb). 1990;48(4):162-175.

15. Townsend P, Phillimore P, Beattie A. Health and deprivation: Inequality and the North, Croom Helm: London, 1987.

16. Choi MH, Yoon TH, Cheong KS, Cho BM, Hwang IK, Kim CH, et al. Deprivation and Mortality at the Town Level in Busan, Korea: An Ecological Study. J Prev Med Public Health 2011;44(6):242-248

17. Cheong KS, Choi MH, Cho BM, Yoon TH, Kim CH, Kin YM, and Hwang IK. Suicide Rate Difference by Sex,

Age, and Urbanicity, and Related Regional Factors in Korea. J Prev Med Public Health 2012;45(2):70-77.

18. Shin YJ, Yoon TH, Kim MH et al. Health promotion strategies and programmes development for health inequalities alleviation. Seoul: Hanyang University & Management Center for Health Promotion; 2009.

19. Pyun HS, Kim KS. The HLM Analysis of Depression and Self-Esteem of the Elderly Living in the Unreported Residential Faclity. Journal of the Korean Gerontological Society 2005;25(4):147~165.

20. Amélie QV. Self-rated health: caught in the crossfire of the quest for 'true' health? Int Jr Epidemiol. 2007;36(6):1161-1164.

21. Heo JH, Cho YT, Kwon SM. The Effects of Socioeconomic Deprivations on Health. Korean journal of sociology 2010;44(2):93-120.

22. Abdel-khalek AM. Happiness, health, and religiosity: Significant relations. Mental Health, Religion & Culture 2006;9(1):85-97.

23. Vera-Villarroel P, Celis-Atenas K, Pavez P, Lillo S, Bello F, Díaz N, López W. Money, Age and Happiness:Association of Subjective Wellbeing with Socio-Demographic Variables. Revista Latinoamericana de Psicología. 2012;44(2):155-163.

24. Choi MO, Moon YS. An Analysis on the Factors Influencing Busan Metropolitan City Residents' Happiness. The Korean Journal of Local Studies 2011;15(1):277-299.

25. Bell D, Blanchflower DG. The Scots may be brave but they are neither healthy nor happy. Scottish Journal of Political Economy 2007;54(2):166-194.

26. Campbell A. Subjective measure of well-being. American Psychologist 1976;31(2):117-124.

27. Diener E, Sandvik E, Seidlitz L & Diener M. The relationship between income and Subjective Well-Being: Relative or Absolute? Social Indicators Research 1993;28:195-223.

28. Do JS. The Effects of Youth Voluntary Service on Subjective Happiness. Journal of Adolescent welfare 2011;13(4):21-46.

29. Kim HS, Choi JH. Korean's Group Activities and the Subjective Well-Being. Korean Journal of Culture and Social Issues 1997;3(1):41-60.

30. Mancini JA, Orthner DK. Situational influences on leisure satisfaction and moral in old age. Journal of the American Geriatrics Society 1980;28(10):466-471.
